# Fast Model for Evaluation of the Thyroid Dosimetry During Chest Tumor Radiotherapy

**DOI:** 10.1177/1559325819889152

**Published:** 2019-11-22

**Authors:** Yiling Wang, Min Zheng, Ling He, Jinhui Xu, Gang Yin, Jie Zhou, Yue Zhao, Ming Jiang, Jie Wang

**Affiliations:** 1 Sichuan Cancer Hospital & Institute, Sichuan Cancer Center, School of Medicine, University of Electronic Science and Technology of China, Chengdu, China; 2 Sichuan Center for Disease Control and Prevention, Chengdu, China; 3 School of Electronic Science and Engineering, University of Electronic Science and Technology of China, Chengdu, China

**Keywords:** thyroid absorbed dose, radiotherapy, chest tumor, radiation protection

## Abstract

Due to the reported high incidence of thyroid cancer induced by radiotherapy, dose assessment is significant to prevent thyroid late effects. Thyroid dosimetry can be evaluated either by entrance skin dose (ESD) measured with thermoluminescent dosimeter (TLD) arrays or by absorbed dose (AD) computed with treatment planning system. However, their correlation has hardly been reported in any publications. Moreover, the reported measurement procedures for thyroid ESD are usually inefficient. This study aims to provide a fast model for efficient acquisition of thyroid ESD and analyze the coherent relationship between ESD and AD. We conducted the study on the China radiation anthropomorphic phantom with intentionally delineated cancers, irradiated by a Varian 23EX linac. We have measured the ESD with TLD at 5 different points, while computed AD with the Oncentra Masterplan TPS. The ESD at the middle gorge of thyroid has exhibited significant linear correlation with those measured at other points. Furthermore, a regressive model has been proposed to predict thyroid AD from ESD. Consequently, it is recommended to only measure the ESD at the middle gorge of thyroid for an efficient dose assessment. The validity of the regressive model to predict thyroid AD from ESD has also been demonstrated.

## Introduction

Being easily exposed to the secondary X-ray of radiotherapy, thyroid gland could be susceptible for irradiation. The post-radiotherapy disorder and dysfunction could be hypothyroidism, autoimmune thyroiditis, ophthalmopathy, and thyroid cancers.^1,2^ The incidence of thyroid cancer could be 7.5 of 1 million cases per 1 cGy absorbed dose (AD), while the female was more susceptible than male.^3^ A high incidence of hypothyroidism was also reported for volume AD exceeding 26 Gy.^4^ Furthermore, volume AD at 2.25 Gy could also lead to follicular cell and vascular damages,^4^ which could induce thyroid dysfunction.^5^

For head and neck cancer, the radiation-induced hypothyroidism incidence rate could be 20% to 40%.^2,6,7^ The thyroid gland was tend to be irradiated at high-dose level, since the planning target volume (PTV) might cover most parts of the thyroid. As for breast cancer, the incidence rate of hypothyroidism was reported to be 6%,^8^ which was much lower than that of the head and neck cancer. However, compared with patients with nonirradiated breast cancer, those who had supraclavicular lymph nodes irradiated were more likely to develop hypothyroidism.^9^ According to a 7-year follow-up, the thyroid hypofunction rate was 21% for patients who had received postoperative locoregional radiotherapy.^10^

The thyroid dosimetry could be evaluated with the entrance skin dose (ESD). However, the reported measurement procedures were usually inefficient, where 85 points could be involved for measurement.^11^ The thyroid dosimetry could also be evaluated with AD simulated in treatment planning system (TPS).^2,12,13^ Nevertheless, uncertainties related to beam modeling and intrafraction management might lead to inaccuracies in dose delivery, making the dose assessment inaccurate. To our best knowledge, the relationship between the measured ESD and simulated AD has not been identified. Furthermore, few works have concerned the efficiency of dose measurement, which was significant for massive data acquisition.

The purpose of this study is to provide a fast model for efficient acquisition of thyroid ESD and analyze the coherent relationship between ESD and AD.

## Materials and Methods

**Figure 1.**
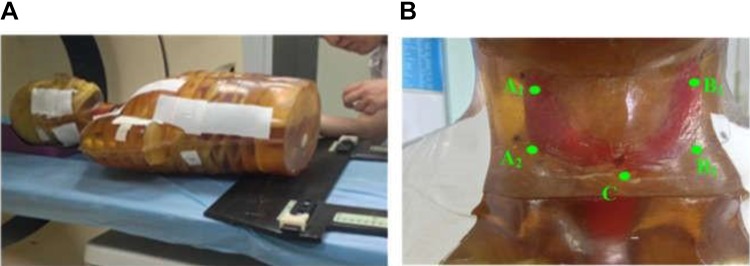
Evaluation of the thyroid ESD on the Chengdu dosimetric phantom. (A) Linac radiotherapy for the phantom. (B) Five points on the thyroid gland for ESD measurement, including the right upper pole A1, the right lower pole A2, the left upper pole B1, the left lower pole B2, and the middle gorge of the thyroid gland C. ESD indicates entrance skin dose.

**Figure 2.**
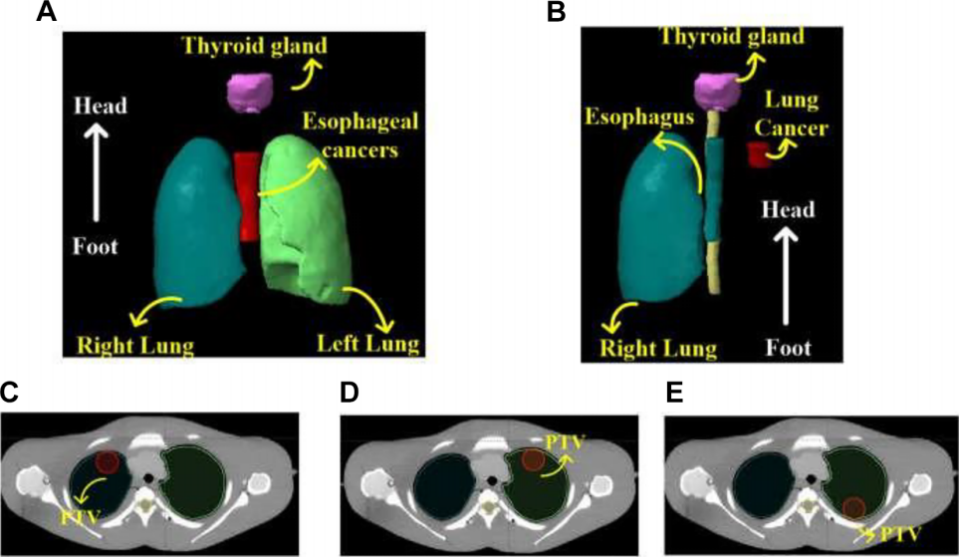
Planning target volume illustration of chest tumor. (A) Esophageal cancer. (B) Lung cancer. (C) Lung cancer at the right anterior of the chest wall in case D. (D) Lung cancer at the left anterior of the chest wall in case E. (E) Lung cancer at the left posterior of the chest wall in case F. PTV indicates planning target volume.

This study was conducted on the China radiation anthropomorphic phantom,^14,15^ as displayed in Figure 1. We intentionally delineated 3 different sized esophagus cancers and 3 different located lung cancers for imitation of PTV. Figure 2A illustrates the esophagus cancers, which were distributed along the mediastinum with fixed distance 5.4 cm from the lower edge of thyroid gland. Their lengths in the head and foot direction were 9 cm in case A, 11 cm in case B, and 14 cm in case C. Figure 2B to E corresponds with the lung cancers, which were located at the right anterior of the chest wall in case D, left anterior of the chest wall in case E, and left posterior of the chest wall in case F. The sizes of PTV were 46.28, 55.91, and 72.87 ccm for cases A to C, and 20.70 ccm in cases D for F, with ccm indicating cubic centimeter.

The 6 MV photon beam radiotherapy was delivered with nominal dose rates (600 MU/min). Specifically, the 2-dimensional conformal radiotherapy (2D-CRT) with 2 static beams and the 3-dimensional CRT (3D-CRT) with 5 static beams were delivered with 1 Varian 23EX linac (Palo alto, California). The intensity-modulated radiotherapy (IMRT)^16^ with 5 static beams and the volumetric modulated arc therapy (VMAT)^17^ were delivered with 1 Elekta Axesse linac (Stockholm, Sweden). The prescription was 60 Gy in 30 fractions delivered to the PTV with 100% of the volume covered by ≥95% of the prescribed dose. The maximal point dose was kept ≤110%. The detailed parameters of external beam irradiation are summarized in Table 1.

**Table 1.** Parameters for External Beam Irradiation.

Case	Therapy	Linac	Gantry Angles (°)	SAD (cm)	Prescription (Gy/F)
A	2D-CRT 3D-CRT IMRT VMAT	Varian 23EX Elekta Axesse	0, 180 210, 320, 0, 40, 150 181-179 two arcs		
B	2D-CRT 3D-CRT IMRT VMAT	Varian 23EX Elekta Axesse	0, 180 210, 320, 0, 40, 150 181-179 two arcs		
C	2D-CRT 3D-CRT IMRT VMAT	Varian 23EX Elekta Axesse	0, 180 210, 320, 0, 40, 150 181-179 two arcs		
D	2D-CRT 3D-CRT IMRT VMAT	Varian 23EX Elekta Axesse	0, 180 200, 320, 0, 40, 160 182-22 two arcs	100	60/30
E	2D-CRT 3D-CRT IMRT VMAT	Varian 23EX Elekta Axesse	0, 180 200, 330, 0, 30, 160 181-71 two arcs		
F	2D-CRT 3D-CRT IMRT VMAT	Varian 23EX Elekta Axesse	0, 180 181, 200, 330, 20, 160 181-177 two arcs		

Abbreviations: 2D-CRT, 2-dimensional conformal radiotherapy; 3D-CRT, 3-dimensional conformal radiotherapy; IMRT, intensity-modulated radiotherapy; SAD, surface axis distance.

We have obtained the thyroid ESD with the precalibrated thermoluminescent dosimeters (TLDs; chips: LiF [Mg, Cu, P], TLD reader analyzer system: RGD-3B), where 5 points were measured, including the right upper pole A_1_, the right lower pole A_2_, the left upper pole B_1_, the left lower pole B_2_, and the middle gorge C of the thyroid gland illustrated in Figure 1B. The accuracy of the TLD was certificated by the China National Institute of Metrology within the range of ±3.1%. We have repeated the measurements for 3 times at each point.

The thyroid AD was computed by the Oncentra Masterplan TPS, involving several organs at risk (OARs) with the same planning constraints in IMRT and VMAT. Considering the thyroid was conventionally not included in chest tumor radiotherapy, no planning constraint was given to thyroid in this study. All the concerned OARs were delineated by an experienced oncologist. We conducted the data analysis with Matlab R2015a (MathWorks, Inc., Natick, Massachusetts).

## Results

### Thyroid ESD and AD of Esophagus Cancer

**Figure 3.**
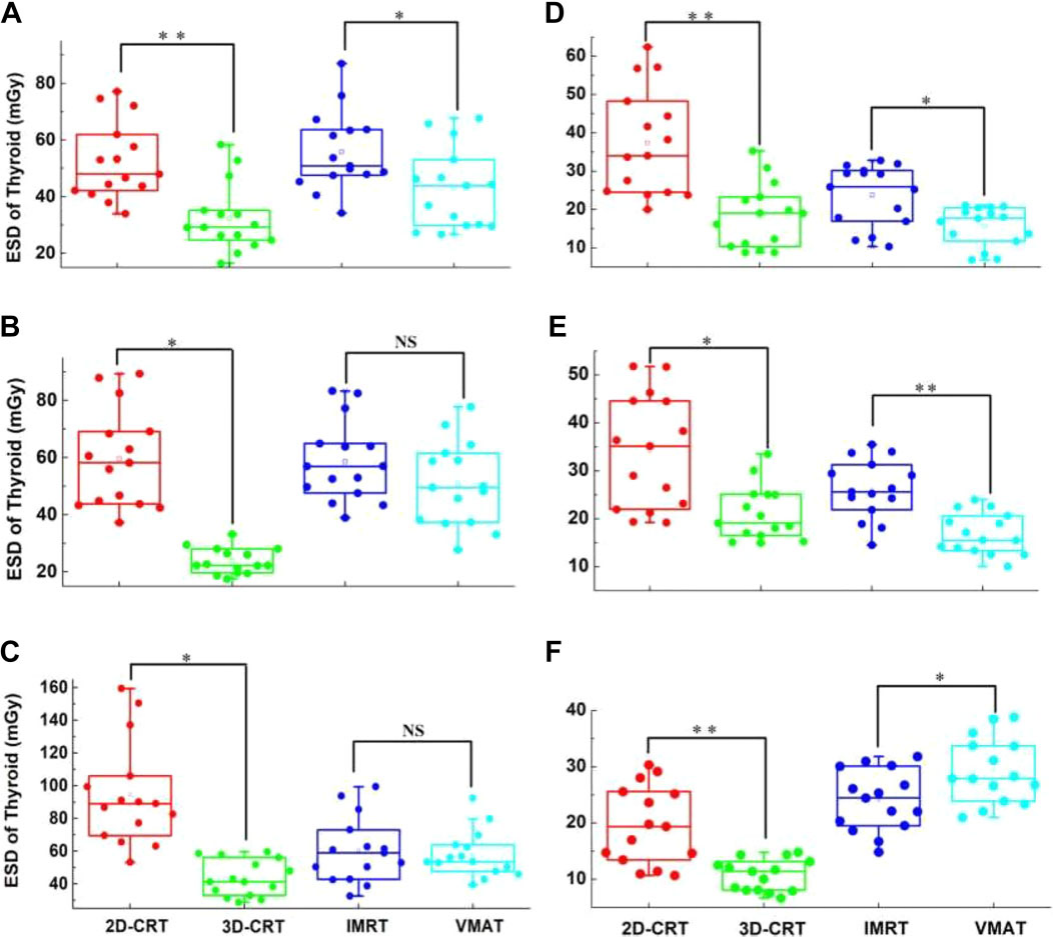
Thyroid ESD results. For each case, the 4 therapy techniques were measured 3 times, respectively. The significance test was conducted for the forward intensity-modulated radiotherapy (2D-CRT, 3D-CRT) and IMRT and VMAT, respectively, where the *P* values were computed by 2-sided Mann-Whitney *U* test. (A) Case A, **P < .001, *P < .05. (B) Case B, *P < 10^−5^, NS, *P* = .171. (C) Case C, *P < 10^−5^, *P* = .934. (D) Case D, **P < .001, *P < .05. (E) Case E, **P < .001, *P < .01. (F) Case F, **P < .001, *P < .05. ESD indicates entrance skin dose; IMRT, intensity-modulated radiotherapy; NS, nonsignificant; VMAT, volumetric modulated arc therapy; 2D-CRT, 2-dimensional conformal radiotherapy; 3D-CRT, 3-dimensional conformal radiotherapy.

For esophagus cancer (cases A, B, and C), the measured ESDs of each radiotherapy techniques in one fraction are displayed in Figure 3 and Table 2. The average thyroid ESD of each radiotherapy technique is also displayed in Table 2.

**Table 2.** Entrance Skin Dose and AD of the Thyroid Gland in 30 Fractions.

Case	Therapy	ESD at C (Gy)	Average ESD (Gy)	Mean AD (Gy)	Volume AD (Gy·ccm)	AD 1 ccm (Gy)
A	2D-CRT 3D-CRT IMRT VMAT	2.23 ± 0.07 1.58 ± 0.16 2.29 ± 0.30 1.96 ± 0.08	1.57 ± 0.07 0.97 ± 0.09 1.68 ± 0.16 1.30 ± 0.04	0.64 ± 0.06 0.20 ± 0.03 0.21 ± 0.05 0.18 ± 0.03	31.7 ± 3.0 9.90 ± 1.5 10.40 ± 2.5 8.90 ± 1.5	0.73 0.28 0.31 0.27
B	2D-CRT 3D-CRT IMRT VMAT	2.60 ± 0.11 0.91 ± 0.08 2.40 ± 0.10 2.11 ± 0.24	1.79 ± 0.04 0.71 ± 0.01 1.76 ± 0.02 1.52 ± 0.06	1.03 ± 0.28 0.44 ± 0.15 0.38 ± 0.12 0.40 ± 0.13	51.10 ± 13.9 21.80 ± 7.4 18.80 ± 5.9 19.80 ± 6.4	1.82 0.89 0.75 0.73
C	2D-CRT 3D-CRT IMRT VMAT	4.47 ± 0.34 1.63 ± 0.17 2.79 ± 0.21 2.36 ± 0.43	2.84 ± 0.11 1.31 ± 0.07 1.81 ± 0.07 1.74 ± 0.14	1.13 ± 0.32 0.37 ± 0.11 0.43 ± 0.13 0.46 ± 0.14	56.02 ± 15.9 18.31 ± 5.5 21.32 ± 6.4 22.80 ± 6.9	2.01 0.69 0.82 0.88
D	2D-CRT 3D-CRT IMRT VMAT	1.27 ± 0.11 0.89 ± 0.13 0.98 ± 0.10 0.57 ± 0.07	1.02 ± 0.02 0.68 ± 0.02 0.78 ± 0.04 0.51 ± 0.03	0.25 ± 0.11 0.17 ± 0.04 0.14 ± 0.03 0.21 ± 0.05	12.43 ± 5.4 8.43 ± 1.98 6.94 ± 1.48 10.41 ± 2.97	0.51 0.28 0.23 0.37
E	2D-CRT 3D-CRT IMRT VMAT	0.74 ± 0.03 0.41 ± 0.03 0.93 ± 0.03 1.1 ± 0.04	0.59 ± 0.02 0.32 ± 0.01 0.72 ± 0.04 0.88 ± 0.01	0.24 ± 0.09 0.14 ± 0.04 0.12 ± 0.03 0.21 ± 0.05	11.89 ± 4.46 6.94 ± 1.98 5.95 ± 1.49 10.41 ± 2.48	0.45 0.25 0.20 0.33
F	2D-CRT 3D-CRT IMRT VMAT	1.31 ± 0.15 0.55 ± 0.06 0.81 ± 0.07 0.59 ± 0.07	1.12 ± 0.04 0.55 ± 0.04 0.71 ± 0.03 0.47 ± 0.02	0.19 ± 0.07 0.16 ± 0.04 0.13 ± 0.03 0.18 ± 0.04	9.42 ± 3.47 7.93 ± 1.98 6.44 ± 1.48 8.92 ± 1.98	0.40 0.27 0.22 0.29

Abbreviations: AD, absorbed dose; 2D-CRT, 2-dimensional conformal radiotherapy; 3D-CRT, 3-dimensional conformal radiotherapy; ESD, entrance skin dose; IMRT, intensity-modulated radiotherapy; VMAT, volumetric modulated arc therapy.

**Figure 4.**
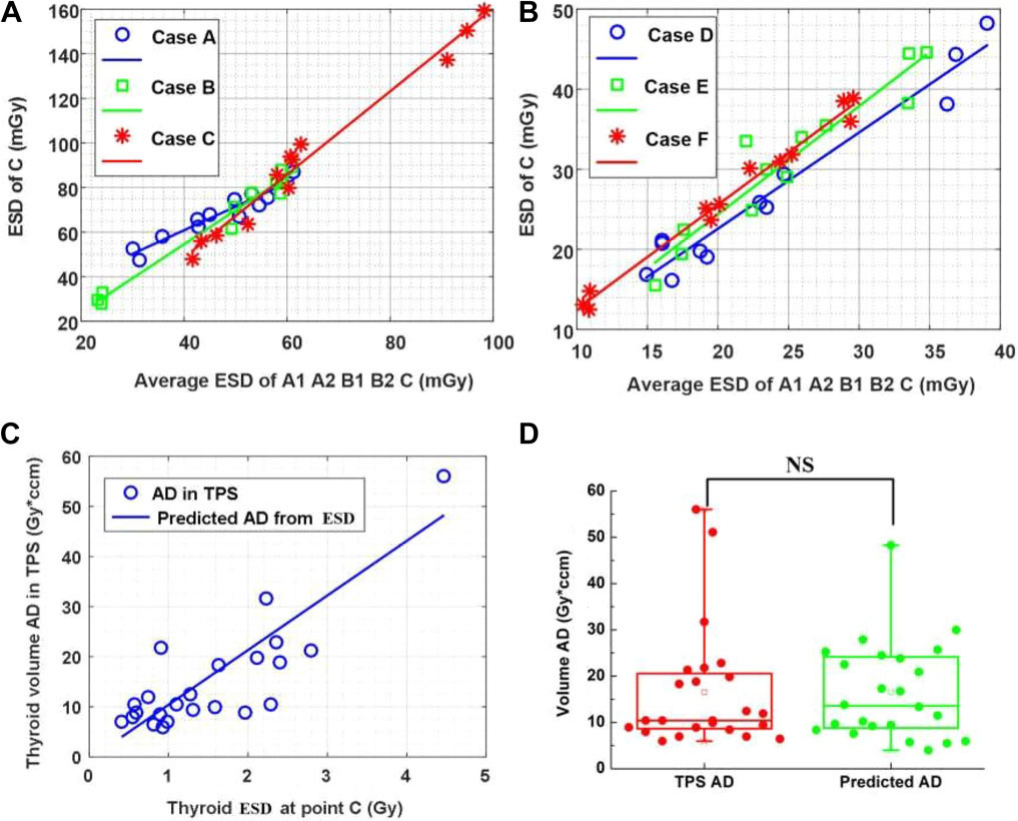
Linear correlation analysis for thyroid ESD at point C, average value of ESD, and volume AD. The *P* values were computed by 2-sided Mann-Whitney *U* test. (A) Linear regression results of the thyroid ESD at point C and the average ESD values of the 5 measured points for cases A to C. (B) Linear regression results of the thyroid ESD at point C and the average ESD values of the 5 measured points for cases D to F. (C) Linear regression relationship between thyroid ESD at point C and the volume AD simulated in TPS, *y* = 10.91*x*-0.50. (D) Comparison of the thyroid volume AD predicted from the ESD at point C and that computed by TPS, *P* = .7493. AD indicates absorbed dose; ESD, entrance skin dose; NS, nonsignificant; TPS, treatment planning system.

Besides, correlation analysis was conducted between the ESD at point C and the average value of the other points, where significant linear correlation was found for case A (*R* ^2^ = 0.951, *P* < .001), case B (*R* ^2^ = 0.903, *P* < .001), and case C (*R* ^2^ = 0.987, *P* < .001). Figure 4A demonstrates the corresponding regression analysis with each dot indicating a certain radiotherapy technique.

The results of TPS simulation were summarized. For both lungs, V_500 cGy_ ≤ 29.1% in case A, V_500_ cGy ≤ 38.0% in case B, and V_500 cGy_ ≤ 40.0% in case C. For heart, V_3000 cGy_ ≤ 6% in case A, V_3000 cGy_ ≤ 24.0% in case B, and V_3000 cGy_ ≤ 32.5% in case C. Table 2 summarizes the thyroid AD computed with TPS.

### Thyroid ESD and AD of Lung Cancer

For lung cancer (cases D, E, and F), the measured ESDs are also demonstrated in Figure 3 and Table 2. Correlation analysis was also conducted between the ESD at point C and the average value of the other points, exhibiting significant linear correlation in case D (*R* ^2^ = 0.950, *P* < .001), case E (*R* ^2^ = 0.993, *P* < .001), and case F (*R* ^2^ = 0.975, *P* < .001), as is shown in Figure 4B.

The results of TPS simulation were also summarized. For both lungs, V_500 cGy_ ≤ 18.2% in case D, V_500 cGy_ ≤ 23.3.0% in case E, and V_500 cGy_ ≤ 22.2.0% in case F. For heart, V_3000 cGy_ ≤ 2.22% in case D, V_3000 cGy_ ≤ 2.42% in case E, and V_3000 cGy_ ≤ 2.22% in case F. Again, Table 2 summarizes the thyroid AD computed with TPS.

## Discussion

The ESD and AD of the thyroid gland for the radiotherapy of chest tumor were evaluated in this study. It was validated that the ESD at the middle gorge of thyroid (point C) has a strong linear correlation with that of other measured points, making it possible to characterize the ESD of thyroid. Therefore, the measurement of ESD at point C was recommended to improve the efficiency.

Besides, we also analyzed the correlative relationship between the measured ESD and the simulated AD. The thyroid ESD was higher than the mean value of AD, which was consistent with the results in reference (11). The attenuation of machine leakage and low-energy scattering radiation might account for it. Furthermore, we also attempted to predict thyroid volume AD from ESD with a linear regression model, where significant correlation was identified (*R* ^2^ = 0.636, *P* < 10^−5^), as illustrated in Figure 4C. Consequently, for each ESD at point C, the corresponding volume AD could be regressively predicted. Finally, the 2-sided Mann-Whitney *U* test was applied to verify the difference between the predicted ADs and the ADs simulated in TPS, exhibiting nonsignificant correlation in Figure 4D.

Furthermore, even the PTV was of small size (20.7-72.9 ccm), the thyroid gland absorbed non-negligible amount of volume dose (all exceeded 2.25 Gy, some even exceeded 26 Gy), which could possibly induce thyroid disorder and dysfunction.^4^ In this study, the thyroid was at a size of 49.57 ccm. With larger size, a higher volume AD should be expected. Besides, different clinical therapy techniques led to different thyroid dosimetry. The 2D-CRT achieved the highest ESD and AD in most cases, due to the fact that the dose distribution of 2D-CRT was less target conformal. Compared with IMRT or VMAT, 3D-CRT was verified to deliver less MUs and had more homogenous dose distribution within the PTV.^18^ Therefore, the thyroid dosimetry of 3D-CRT was the lowest in some cases. Besides, compared with IMRT, VMAT achieved lower thyroid dosimetry in most cases, owing to a more favorable dose distribution. However, case F was the exception. Since the thyroid gland was not involved in the treatment planning process, the related dose distribution could be less favorable from a 360° beam irradiation.

Finally, some problems remained. It should be noted that neither of the 2 dose evaluation schemes in this study was sufficient to quantify the thyroid AD, since the ESD could only measure the dose at the skin, while dose discrepancy related to beam modeling and intrafraction management existed in TPS-simulated AD. Subsequent studies for a specially designed phantom, where measurement instrument could be placed exactly at the thyroid, were required to address the problem. Besides, to clinically determine the thyroid late effects of chest tumor radiotherapy, subsequent studies including surveillance of the thyroid function were required.

## Conclusion

This study has evaluated the correlation between thyroid ESD and thyroid AD in chest tumor radiotherapy. Strong linear correlation has been observed between the ESD at the middle gorge of thyroid gland and the other measured points. Therefore, to improve the efficiency of ESD measurement, it is suggested to only measure the ESD at the middle gorge of thyroid. Besides, the validity of the regressive model to predict thyroid AD from ESD has also been demonstrated. Furthermore, it should be clinically concerned that the thyroid gland could receive a non-negligible amount of scattering irradiation during the radiotherapy of chest tumor, inducing the potential risk of thyroid disorder and dysfunction. To protect the thyroid, it is suggested to shield the thyroid gland during the radiotherapy of chest tumor.
